# The Relation Between Capillary Transit Times and Hemoglobin Saturation Heterogeneity. Part 2: Capillary Networks

**DOI:** 10.3389/fphys.2018.01296

**Published:** 2018-09-21

**Authors:** Adrien Lücker, Timothy W. Secomb, Matthew J. P. Barrett, Bruno Weber, Patrick Jenny

**Affiliations:** ^1^Department of Mechanical and Process Engineering, Institute of Fluid Dynamics, ETH Zürich, Zurich, Switzerland; ^2^Department of Physiology, University of Arizona, Tucson, AZ, United States; ^3^Institute of Pharmacology and Toxicology, University of Zürich, Zurich, Switzerland

**Keywords:** blood flow, capillary transit time heterogeneity, computational modeling, hematocrit, hemoglobin saturation, microcirculation, oxygen transport, red blood cells

## Abstract

Brain metabolism is highly dependent on continuous oxygen supply. Cortical microvascular networks exhibit heterogeneous blood flow, leading to non-uniform tissue oxygenation and capillary hemoglobin saturation. We recently proposed capillary outflow saturation heterogeneity (COSH) to represent effects of heterogeneity on oxygen supply to tissue regions most vulnerable to hypoxia, and showed that diffusive oxygen exchange among red blood cells within capillaries and among capillaries (diffusive interaction) significantly reduces COSH in simplified geometrical configurations. Here, numerical simulations of oxygen transport in capillary network geometries derived from mouse somatosensory cortex are presented. Diffusive interaction was found to reduce COSH by 41 to 62% compared to simulations where diffusive interaction was excluded. Hemoglobin saturation drop across the microvascular network is strongly correlated with red blood cell transit time, but the coefficient of variation of saturation drop is approximately one third lower. Unexpectedly, the radius of the tissue cylinder supplied by a capillary correlates weakly with the anatomical tissue cylinder radius, but strongly with hemoglobin saturation. Thus, diffusive interaction contributes greatly to the microcirculation's ability to achieve tissue oxygenation, despite heterogeneous capillary transit time and hematocrit distribution. These findings provide insight into the effects of cerebral small vessel disease on tissue oxygenation and brain function.

## Introduction

Microvessels actively participate in the oxygen supply to parenchymal cells and hence are fundamental for oxidative energy metabolism. Beyond having a large surface area for gas exchange, they are directly involved in blood flow regulation. In the cerebral cortex, arterioles (Attwell et al., 2010) as well as capillaries (Tian et al., 2010; Hall et al., 2014) are essential components of neurovascular coupling. Their importance implies that malfunctions in the microvasculature may have adverse consequences on healthy brain function (Pantoni, 2010).

At the most fundamental level, oxygen supply to tissue depends on the rate of blood flow per volume of tissue (perfusion) and the oxygen content of the arterial blood, which in turn depends on the hematocrit and the oxygen saturation of hemoglobin (Popel, 1989). Any alteration in these bulk variables can affect tissue oxygenation. However, the distribution of oxygen levels in tissue depends also on the microvascular network structure and flow and hematocrit distributions, which are markedly heterogeneous (Duling and Damon, 1987). Therefore, in analyzing the delivery of oxygen to tissue, it is essential to consider not only the overall oxygen supply but also the impact of heterogeneity in the microcirculation (Ellsworth et al., 1988).

As a measure of this heterogeneity, the capillary transit time heterogeneity (CTH) has been defined as the standard deviation of the transit time distribution of red blood cells (RBCs) through capillaries. Jespersen and Østergaard (2012) showed with a simple theoretical model based on the Bohr-Kety-Crone-Renkin equation that the oxygen extraction fraction decreases with increasing CTH, even if the mean flow stays constant (Jespersen and Østergaard, 2012; Angleys et al., 2015). However, CTH does not take into account effects of variability in path lengths, hematocrit and capillary spacing on tissue oxygenation, or effects of diffusive transfer between tissue territories surrounding capillaries.

Capillary blood oxygen content is lowest at locations most distant from arterioles, making surrounding tissue most vulnerable to hypoxia. In the presence of flow maldistribution, some capillaries may have such low end-capillary Po_2_ that local hypoxia ensues even if the average hemoglobin saturation (HS) is sufficiently high. Therefore, we introduced the concept of capillary outflow saturation heterogeneity (COSH) as a measure of heterogeneity that is directly relevant to oxygen delivery to tissue, and analyzed the factors influencing COSH using theoretical models (Lücker et al., 2018). Using simplified geometries, we showed that CTH, hematocrit heterogeneity and, to a lesser extent, capillary spacing are important sources of COSH, the effects of which are substantially reduced by diffusive interaction. The occurrence of local tissue hypoxia depends both on the average capillary outflow HS and on COSH. For a given average HS, an increase in COSH causes a decrease in the oxygen levels in capillaries with low HS and may lead to an increase in the amount of hypoxic tissue.

The diffusive interaction models introduced in Lücker et al. (2018) assumed either a single capillary surrounded by a cylindrical tissue region or an array of evenly spaced parallel capillaries. In reality, cortical capillary networks (CNs) have a highly interconnected mesh-like structure (Lorthois and Cassot, 2010; Blinder et al., 2013) with tortuous capillaries and irregular spacings. Such geometries can have a major impact on the distribution of tissue Po_2_ levels, including the occurrence of local hypoxic tissue regions that would not be present if the vessels were parallel and evenly spaced with the same overall vascular density (Secomb et al., 2000). Therefore, it is a priori unclear whether the model predictions for capillary diffusive interaction from Lücker et al. (2018) hold for cerebral blood flow in realistic capillary networks. A detailed analysis of COSH in cortical CNs is thus needed.

The main sources of COSH can be identified as: (1) heterogeneous saturation levels entering capillaries; (2) heterogeneous capillary transit times, affecting the time available for oxygen unloading; (3) heterogeneous capillary hematocrit, which affects the net blood oxygen content; (4) heterogeneous capillary spacings, affecting the amount of oxygen consumed per unit length. This work focuses on how diffusive interaction in cortical CNs mitigates the occurrence of COSH resulting from these four sources. Here, COSH is not compared to mean HS since diffusive interaction is not expected to influence the mean HS at the distal end of capillaries (Lücker et al., 2018). Additionally, the effect of CN geometry on tissue oxygenation has been investigated previously (Hsu and Secomb, 1989; Goldman and Popel, 1999, 2000) and is not addressed here.

In this study, we employ numerical simulations to compute HS in two CNs acquired from the mouse somatosensory cortex. To this end, a novel flow reconstruction algorithm based on sparse RBC flow measurements is developed. COSH is evaluated using a computational model with moving RBCs and a simpler differential equation model for HS. This makes it possible to quantify the effect of diffusive interaction on COSH and to estimate the respective influences of diffusive interactions between different capillaries and within single capillaries. Additionally, the relation of HS at the distal end of capillaries with transit times is examined, and the tissue territories supplied by individual capillaries are compared with those defined based on the nearest vessel. Numerical simulations of COSH are an effective tool to investigate how diffusive interaction contributes to the robustness of oxygen delivery from the microcirculation. The balance between the sources of COSH and diffusive interaction may play a central role in cerebral small vessel disease and its consequences on brain function.

## Materials and methods

The effects of RBC and capillary diffusive interactions in CNs are quantified using a computational model with moving RBCs and models based on a differential equation for the HS. The magnitude of both types of diffusive interaction is evaluated by comparing the results produced by these models. The model predictions made in Lücker et al. (2018) are compared to simulated HS distributions in realistic CNs.

### Models based on hemoglobin saturation

The models for the evolution of hemoglobin saturation heterogeneity (HSH) were derived in Lücker et al. (2018). Here only the equations that will be referred to are reproduced. The subscripts *c*, *p*, *w*, and *t* denote the RBCs, the plasma, the capillary endothelium and the tissue, respectively. The proximal and distal ends of capillary segments or whole capillary paths are denoted with the subscripts *a* (for “arterial”) and *v* (for “venous”).

RBCs are modeled as cylinders with volume *V*_rbc_ and radius *r*_*c*_. Blood flow through capillaries is described by RBC velocity *v*_rbc_, tube hematocrit *H*_*T*_ and linear density μ_*LD*_ which is the ratio of the length occupied by RBCs to the total capillary length. The equilibrium curve between hemoglobin and oxygen is modeled using the Hill equation. Its inverse is denoted by *P*_eq_(*S*). The following equations are based on a straight capillary surrounded by an axisymmetric tissue region. The axial and radial coordinates are denoted by *x* and *r*, respectively.

The models for the HS *S* rely on the equation

(1) $$Q_{{\text{O}}_{2}}(S)\frac{\text{d}S}{\text{d}x}=-j_{t}(x).$$

(Equation 10 in Lücker et al., 2018). The convective oxygen carrying capacity is

(2) $$Q_{{\text{O}}_{2}}(S)=v_{\text{rbc}}\left(\mu_{LD}\pi t_{c}^{2}C_{0}+\pi r_{p}^{2}\alpha_{\text{eff}}\frac{\text{d}P_{\text{ep}}}{\text{d}S}\right),$$

where *C*_0_ = *N*_Hb_*V*_mol,O_2__ is the product of the heme concentration and the molar volume of oxygen and α_eff_ is the effective oxygen solubility in the capillary. By neglecting axial diffusion, the local oxygen extraction rate reduces to

(3) $$j_{t}\left(x\right)=M_{0}\pi \left(r_{t}^{2}\left(x\right)-r_{w}^{2}\right),$$

where *M*_0_ is the metabolic oxygen consumption rate per unit volume. The HS after a transit time τ is obtained by integrating Equation (1), which yields

(4) $$S\left(\tau \right)=S_{a}-\int_{0}^{\tau }\frac{j_{t}\left(v_{\text{rbc}}t\right)}{\mu_{LD}\pi r_{c}^{2}C_{0}+\pi r_{p}^{2}\alpha_{\text{eff}}\frac{\text{d}P_{\text{eq}}}{\text{d}S}}\text{d}t.$$

Unlike the idealized geometries used in Lücker et al. (2018), Equation (1) will be integrated in capillary networks by starting from the inflow vessels. At a converging bifurcation of two parent vessels with indices 0 and 1, the HS at the start of the child vessel with index 2 needs to be specified. The distribution of HS in the child vessel will be modeled by integrating Equation (1) separately for both values of *S* from the parent vessels. Namely, we use a discrete distribution (*S*_*v*,0_, *w*_0_), (*S*_*v*,1_, *w*_1_) at the inlet of the child vessel, where *S*_*v,i*_ (*i* = 0, 1) is the distal HS in the respective parent vessel and *w*_*i*_ = *q*_rbc,*i*_/(*q*_rbc,0_ + *q*_rbc,1_), (*i* = 0, 1) are RBC-flow-based weights. This process is repeated at each converging bifurcation. If discrete distributions converge at a node, the respective weights in the child vessel are set similarly by taking the upstream weights into account. With this method, there is no RBC diffusive interaction unlike in the computational method presented below, hence this phenomenon can be isolated. A minimal HS of zero was enforced.

For an array of parallel capillaries, our model for capillary diffusive interaction based on the continuity of tissue Po_2_ results in the following equation for the HS difference Δ*S* between two pairs of capillaries

(5) $$Q_{{\text{O}}_{2}}\left(\bar{S}\right)\frac{\text{d}\Delta S}{\text{d}x}={-\frac{\Delta S}{K_{CI}}\frac{\text{d}P_{\text{eq}}}{\text{d}S}|}_{\bar{S}},$$

where $\bar{S}$ is the HS averaged over the capillaries. The model coefficient *K*_*CI*_ is defined by

(6) $$K_{CI}=K_{IV}+\frac{1}{2\pi D_{t}\alpha_{\text{t}}}\left(\log\left(\frac{r_{t,\text{mean}}}{r_{w}}\right)-\frac{1}{2}\right),$$

where *r*_*t*, mean_ is the average tissue radius of the tissue slice normal to the capillary.

The diffusive interaction between RBCs in a single capillary was modeled by assuming that the oxygen flux out of a RBC is proportional to the Po_2_ difference between the RBC and the tissue. This assumption results in an evolution equation for the standard deviation σ_*S*_ of HS within a capillary:

(7) $$Q_{{\text{O}}_{2}}\left(\bar{S}\right)\frac{\text{d}\sigma_{S}}{\text{d}x}={-\frac{\sigma_{S}}{K_{RI}}\frac{\text{d}P_{\text{eq}}}{\text{d}S}|}_{\bar{S}}.$$

In this context, $\bar{S}$ is the averaged HS in the capillary. Both interaction models (Equations 5, 7) predict an exponential decrease of HSH as a function of path length and transit time.

### Computational model

The HS values produced by the differential Equation (1) are compared to the results from the computational model with moving RBCs (Lücker et al., 2014) which is summarized in Lücker et al. (2018). The average HS in each individual RBC can be extracted at each time step. For the purpose of this study, this computational model was extended to realistic CNs. The RBC trajectories were computed using the discrete RBC model by Schmid et al. (2017). The convective transport term in the oxygen transport equation and the region-dependent coefficients (such as diffusivity and solubility) require special care. Namely, the diffusion and solubility coefficients can take different values in RBCs, the plasma, the capillary endothelium and the tissue. In grid cells that intersect multiple regions, a suitable interpolation of these coefficients needs to be employed. Metabolic oxygen consumption was assumed to be constant in the tissue for consistency with the diffusive interaction models. The model extension is presented in Supplementary Methods 1.

### Capillary networks

**Figure 1:**
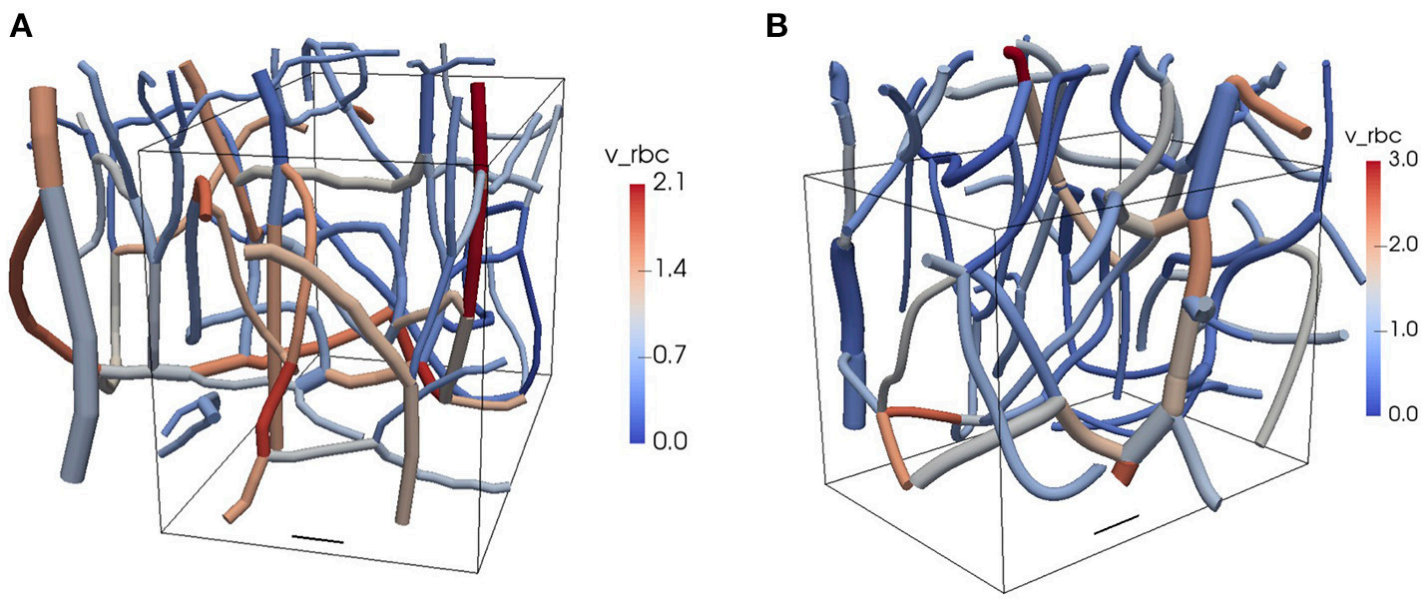
Reconstructed CNs from the mouse cerebral cortex. The colors show the RBC velocities obtained from the flow reconstruction algorithm. The vessel diameters are to scale and the top of the domain is closest to the cortical surface. The boxes show the computational domains used in the oxygen transport simulations. **(A)** CN 1. **(B)** CN 2. Scale bar: 20 μm. v_rbc: RBC velocity. See Table 1 and Figure 2 for further information about the network topology and the reconstructed flow.

We performed simulations in two reconstructed CNs from the mouse somatosensory cortex (Figure 1). The details of the *in vivo* experiments are given below. Two *in vivo* two-photon microscopy datasets were analyzed according to Schneider et al. (2015). Segmentation and centerline extraction was used to reconstruct the CNs. Anisotropic point spread functions, limited spatial resolution and (in the case of *ex vivo* imaging) tissue shrinkage lead to a distortion of the imaging data, which in turn induces spurious metrics. Therefore, vessels are usually assumed to be cylindrical and re-scaling of the vascular calibers is applied. In the present work, the vessel diameter distribution was scaled to a mean of 5 ± 1.5 μm (SD) and a lowest bound of 3 μm was enforced, a distribution chosen to be in concordance with our own previous data (Reichold et al., 2009; Hirsch et al., 2012; Schneider et al., 2015; Schmid et al., 2017) and those from others (Tsai et al., 2009; Blinder et al., 2013; Rungta et al., 2018). It cannot be excluded that some of the acquired vessels were small arterioles or venules. The radii of the RBC and the capillary endothelium were based on proportions similar to those used in Lücker et al. (2017) with plasma radius *r*_*p*_ = 2.0 μm. Therefore, the RBC radius was set to *r*_*c*_ = 0.8*r*_*p*_ and the endothelium radius to *r*_*w*_ = 1.25*r*_*p*_. Additionally, the RBC diameters were clamped between 3 and 8 μm to avoid unrealistic shapes.

In each CN, measurements of RBC velocity were performed in selected vessels as explained below. The flow field in both networks was reconstructed based on the measured RBC velocities and minimization of hydraulic power (see Supplementary Methods 2). A tube hematocrit of 0.25 was set at the inflow vessels. The topology and flow characteristics in both CNs are summarized in Table 1.

**Table 1:** Topology and flow characteristics in both reconstructed CNs (mean ± SD).

	**CN 1**		**CN 2**	
**Parameter**	**Whole**	**Sub-network**	**Whole**	**Sub-network**
Bounding box [μm]	219 × 220 × 168	126 × 216 × 152	194 × 193 × 149	108 × 148 × 116
Vessels	92	60	89	45
Converging bifurcations	22	14	23	14
Diameter [μm]	5.03 ± 1.45	4.91 ± 1.11	5.01 ± 1.47	5.26 ± 1.59
Vessel length [μm]	57.3 ± 41.1	58.1 ± 39.9	47.0 ± 34.5	52.5 ± 37.9
RBC velocity [mm/s]	0.879 ± 0.475	0.899 ± 0.518	0.997 ± 0.564	1.095 ± 0.592
Tube hematocrit	0.221 ± 0.087	0.235 ± 0.085	0.265 ± 0.09	0.254 ± 0.082

*The whole networks were used by the flow reconstruction algorithm and the sub-networks were employed in the oxygen transport simulations*.

### Computation of supplied tissue territories

The tissue territories supplied by each capillary are at the heart of capillary diffusive interaction. The tissue can be subdivided based on either the nearest vessel or on the vessel which it is actually supplied by. For a given vessel, the associated geometric tissue territory consists of all tissue elements that are closest to the vessel. These regions were computed by discretizing the tissue into cubic elements with size 1 μm and assigning them to the vessel with the closest centerline. By assuming a cylindrical shape for the tissue territory with volume *V*_*t,i*_ around the vessel *i*, an associated geometric tissue radius *r*_*t,i*_ can be determined based on

(8) $$V_{t,i}=L_{i}\pi \left(r_{t,i}^{2}-r_{w,i}^{2}\right),$$

where *L*_*i*_ is the length of vessel *i*.

The geometric tissue radii will be compared to the functional tissue radii which are obtained using the actual oxygen extraction from each vessel. For a vessel *i* with HS *S*_*a,i*_ and *S*_*v,i*_ at the proximal and distal ends, respectively, the functional tissue radius *r*_*t,i*_ is computed by fitting the local oxygen extraction rate *j*_*t*_ (Equation 3) such that the integration of Equation (1) along vessel *i* yields the prescribed drop in HS. The associated functional tissue volume is obtained using Equation (8).

The numerical results were controlled by examining the respective sums of geometric and functional tissue volumes. These should be equal, since the total metabolic oxygen consumption in the CNs is proportional to the total tissue volume with the employed models. The discrepancy was smaller than 1.0% in CN 1 (2.0% in CN 2). These slight deviations most probably occur since the RBC flow through the network is permanently fluctuating (Schmid et al., 2017) and hence tissue Po_2_ as well.

### Model parameters

The same physiological parameters as in Lücker et al. (2018, Table 1) were used. At the tissue boundary, the gradient of the Po_2_ field was set to zero. In each RBC entering the domain, HS was set to a constant value within the RBCs. Two ways of setting this value were employed. First, a constant inlet value of *S*_*a*_ = 0.6 was applied at each inflow vessel. Second, a random inlet value uniformly distributed between 0.5 and 0.7 was assigned to each inflow vessel. In each RBC that enters the computational domain, HS was set to the value assigned to the vessel where the RBC is located. These values are in the range computed by Sakadžić et al. (2014) based on Po_2_ measurements in the rodent cerebral cortex. The grid spacing was set to 1 μm in the tissue domain and to 0.67 μm for RBCs with a diameter of 4 μm. The mesh size was 126 × 216 × 152 in CN 1 and 108 × 148 × 116 in CN 2. The time step was set to 1 ms.

### Experimental procedures

Two adult, female C57BL/6J mice (Charles River, Germany) were used for these experiments. The mice were prepared for chronic imaging as described previously (Mächler et al., 2016; Stobart et al., 2016).

Imaging data were acquired using a custom built two-photon microscope (Mayrhofer et al., 2015). For imaging sessions, the mice were anesthetized subcutaneously with a mixture of fentanyl (0.05 mg/kg, Sintenyl, Sintetica), midazolam (5 mg/kg, Dormicum, Roche), and medetomidine (0.5 mg/kg, Domitor, Orion Pharma), and anesthesia was maintained with midazolam (5 mg/kg) after 50 min. The vasculature was labeled using FITC dextran (5%, 59–77 kDa, Sigma), injected via the tail vein.

High resolution structural images of microvasculature networks within the somatosensory cortex were obtained at an in-plane resolution of ~ 0.4 × 0.4 μm, and spaced ~2 μm apart in depth. Both networks were located in the upper 250 μm of the cortex.

RBC velocities in individual vessels were measured using line scans along the axis of the vessel (Kleinfeld et al., 1998). Briefly, the RBCs appear as dark shadows compared to the fluorescently-labeled plasma, and their motion along the vessel leads to streaks on a space-time image. The angle of these streaks can be used to calculate RBC velocity, which was done here using custom-built software based on an algorithm using the Radon transform (Drew et al., 2010).

All procedures involving animals were approved by the Canton of Zurich Veterinary Office, in accordance with Swiss law (Federal Act of Animal Protection 2005 and Animal Protection Ordinance 2008).

## Results

**Figure 2:**
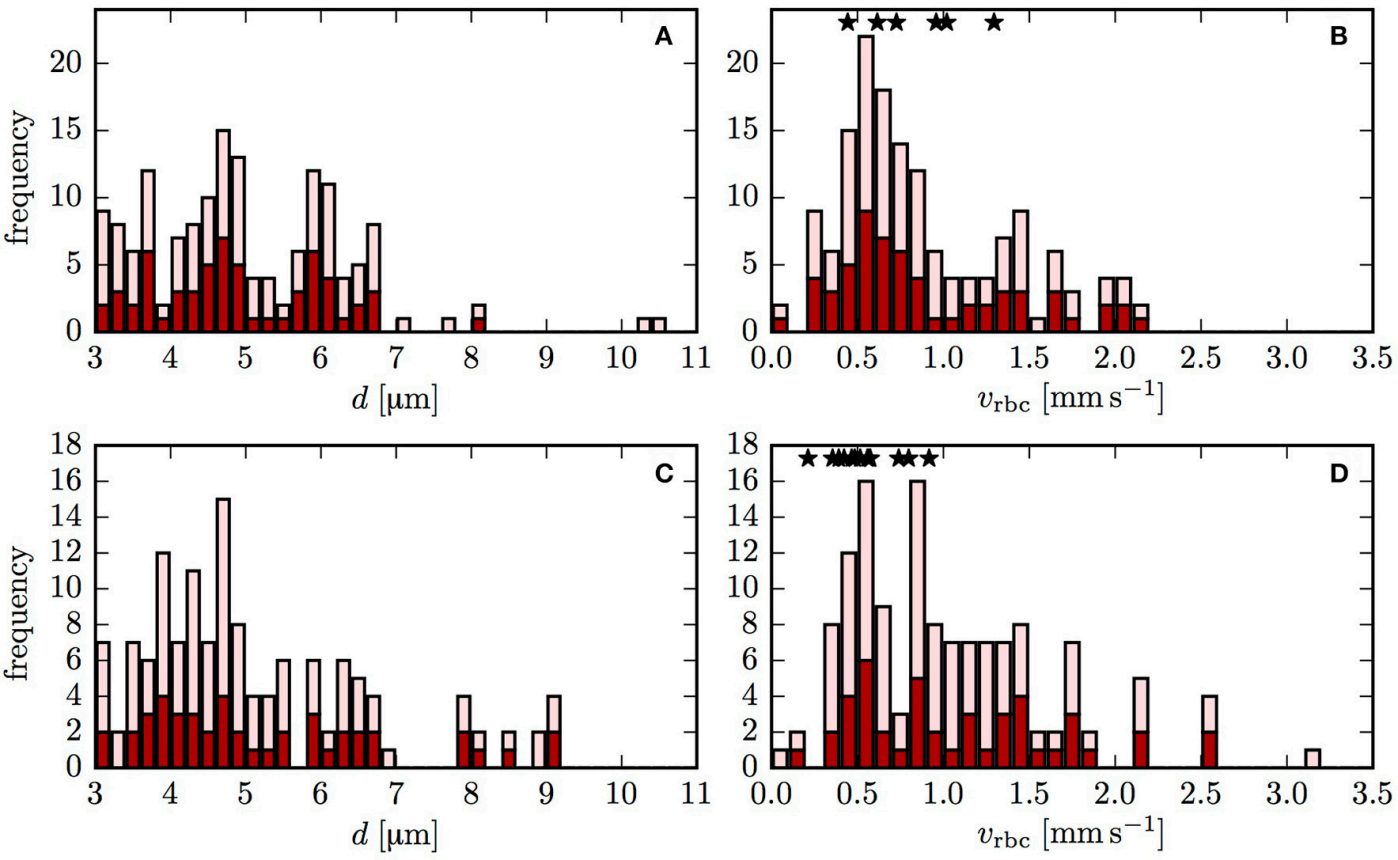
Vessel diameters and RBC velocities in both reconstructed CNs. The dark red bars indicate the vessels that were included in the oxygen transport simulation. The light red bars refer to the vessels that were used in the flow reconstruction, but not in the oxygen transport simulations. The stars show the experimentally measured values of RBC velocity. **(A,B)** CN 1; **(C,D)** CN 2.

HS was simulated in two CNs using the computational model with moving RBCs and a differential equation model (Equation 1). The blood flow in both CNs was reconstructed based on flow measurements as explained in the Supplementary Methods 2. Figure 2 shows the diameters and the RBC velocities obtained using the flow reconstruction algorithm. Since there were no available Po_2_ measurements, a boundary value for HS for RBCs entering the computational domain needs to be set. As explained above, two boundary conditions (constant inflow HS and random HS values assigned to each inflow capillary) were used to assess whether the results are independent of the choice of boundary conditions. With the former, HSH develops only inside the CNs, partly due to heterogeneous RBC transit times. The latter directly introduces HSH into the computational domain.

The simulations based on the computational model with moving RBCs were run for 10 s to allow the system to reach a statistically steady state. All shown results were averaged over the last 2 s of simulation time. Supplementary Video 1 shows an oxygen transport simulation in CN 1. The differential equation based model (Equation 1) was also integrated in both CNs starting from the inflow vessels. The values of *v*_rbc_ and μ_*LD*_ required by this model were averaged over the same time window. The local oxygen extraction rate *j*_*t*_ (Equation 3) was evaluated using either the geometric or functional tissue radii that were computed as explained above. The respective influence of RBC diffusive interaction and capillary diffusive interaction can be quantified based on these models.

**Figure 3:**
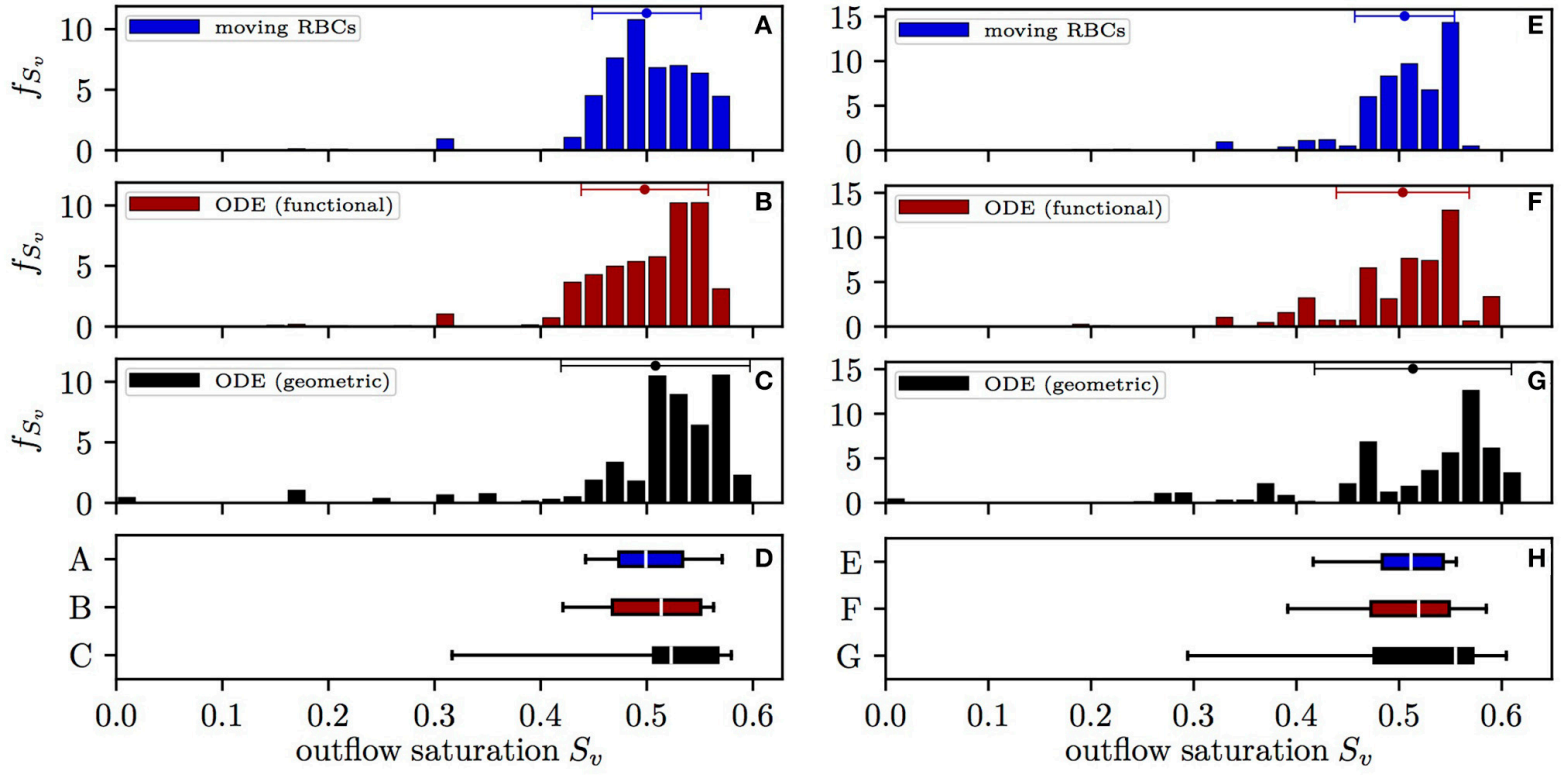
Distribution of the HS *S*_*v*_ at the distal end of capillary paths in CN 1. Left: constant inflow value; right: random inflow value. **(A,E)** Computational model with moving RBCs; **(B,F)** differential equation model with functional tissue radii; **(C,G)** differential equation model with geometric tissue radii; **(D,H)** box plot with whiskers for the 5th and 95th percentile. The values from the differential equation model are weighted by the RBC flow in the distal capillaries. Error bars above the histograms: mean ± SD.

The distribution of the HS *S*_*v*_ at the distal end of capillary paths in CN 1 obtained with the different models is shown in Figure 3. Here, values are expressed as mean ± SD for the simulation with constant inflow value, unless otherwise stated. With the moving RBC model, the distal HS (0.500±0.0512) has lowest standard deviation. The differential equation model with functional radii (which does not take into account RBC diffusive interaction) yields a slightly higher standard deviation (0.498±0.0599). Finally, the differential equation model with geometric radii, where both types of diffusive interaction are absent, results in the largest spread of *S*_*v*_ (0.508±0.0891). Therefore, diffusive interaction causes a 42% reduction of COSH with constant inflow HS. This reduction has a similar magnitude (50%) in the simulations with random inflow HS. Without diffusive interaction, 2.9% of the RBCs have a HS lower than 0.2 upon leaving the CN while this fraction is only 0.3% with the moving RBC model. The mean value of *S*_*v*_ is slightly higher when integrating the differential equation model with geometric radii since a non-negligible fraction of RBCs reaches zero HS and thus no longer supplies the surrounding tissue territories with oxygen. The results obtained in CN 2 are similar, albeit less RBCs reach a low HS due to the smaller network size (Figure S1). Thus, diffusive interaction substantially reduces the heterogeneity of HS at the distal end of capillaries and thereby reduces the quantity of RBCs with low HS.

**Figure 4:**
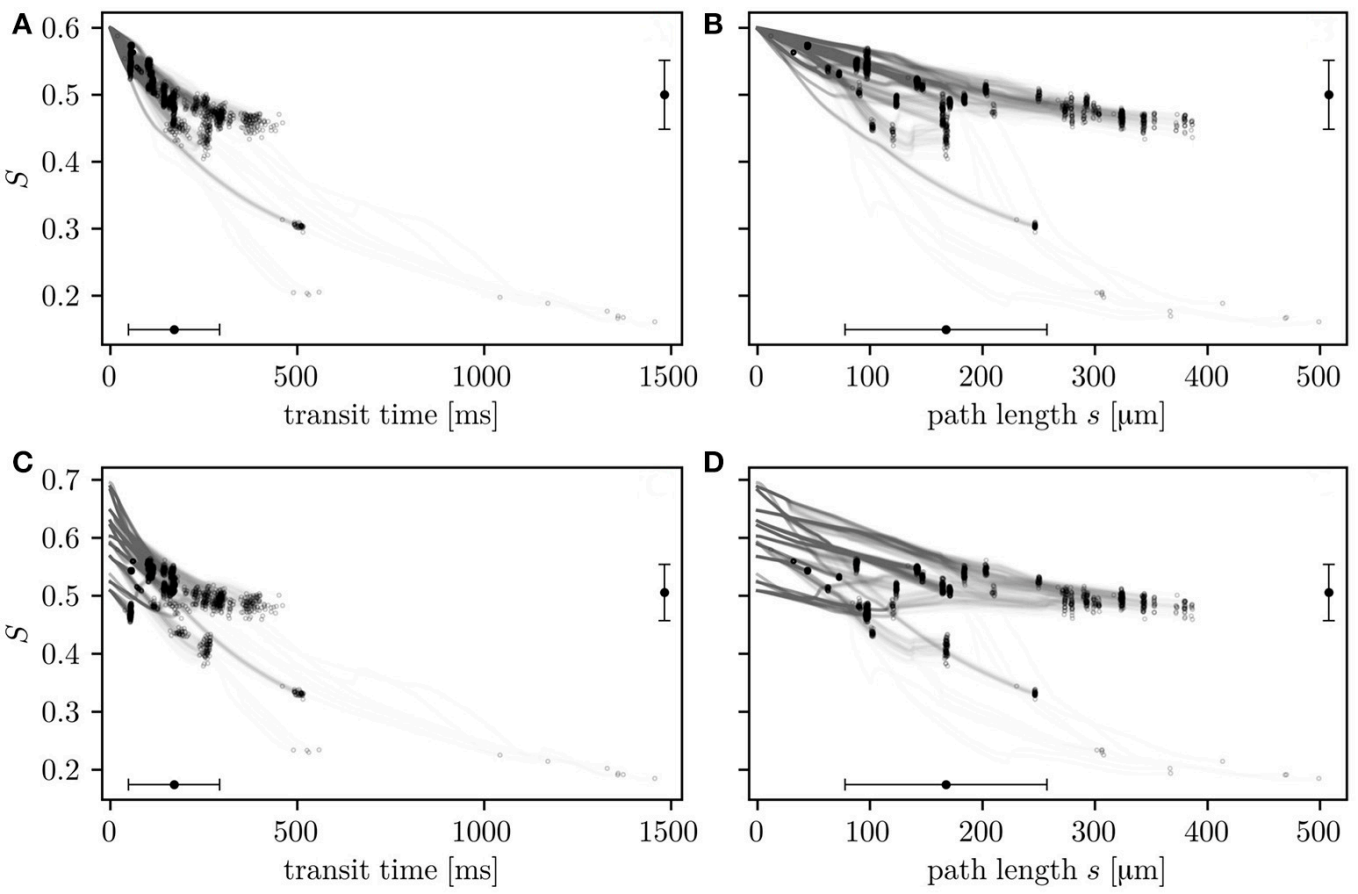
HS along individual RBC paths as a function of transit times **(A,C)** and transit path lengths **(B,D)**. The values are shown for simulations in CN 1 with constant **(A,B)** and random **(C,D)** inflow values. Circles: value in RBCs upon leaving the CN. Error bars: mean ± SD. The horizontal error bars pertain to entire RBC paths through the CN and the vertical error bars to outflow HS.

The relation of HS with transit times and path lengths is now examined (Figure 4). After a given transit time or path length, HS values are heterogeneous. This reflects that *S* is not only determined by the transit time, but also hematocrit and the local oxygen extraction rate (Equation 4) which are heterogeneous quantities in the investigated CNs. Consistently with Equation (4), the HS drop across the network correlates better with transit times (Pearson's correlation coefficient *r* = 0.88 in CN 1, *r* = 0.81 in CN 2) than transit path lengths (*r* = 0.64 in CN 1, *r* = 0.61 in CN 2). The simulations with constant inflow value for *S* allow the comparison of the HSH that develops within the network with the transit time heterogeneity. The HS drop Δ*S* along whole RBC paths has a coefficient of variation of 0.515 in CN 1 (Δ*S* = 0.0993±0.0512). It is 28% lower than that of transit times which is 0.712 (transit time = 0.171±0.122 s). The trends are similar in CN 2 (Figure S2), where the coefficient of variation of the HS is 37% lower than that of the transit times. In the simulations with random inflow values, RBCs with higher inflow HS release more oxygen over their paths, as well as per unit time and length (Figure S3). These results quantitatively illustrate how diffusive interaction compensates the HSH caused by the variability in transit times, hematocrit and inflow saturation.

**Figure 5:**
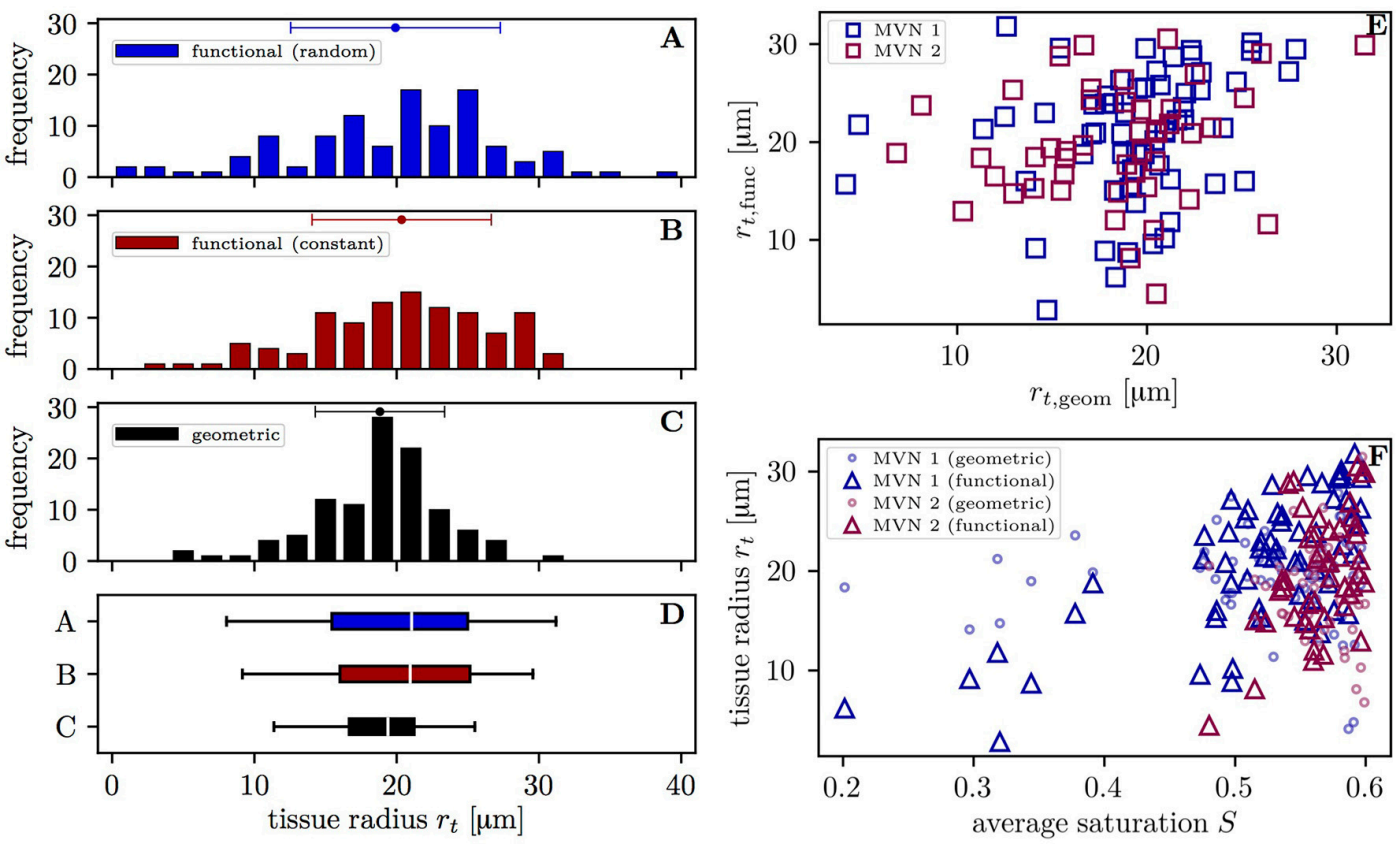
Distribution of the tissue radii in both CNs. Left: histograms of the tissue radii for each vessel in both CNs; **(A)** functional tissue radii with random inflow value; **(B)** functional tissue radii with constant inflow value; **(C)** geometric tissue radii; **(D)** box plot with whiskers for the 5 and 95th percentiles. Error bars above the histograms: mean ± SD. Right: scatter plots of the tissue radii for each vessel; **(E)** functional against geometric tissue radii; **(F)** tissue radii against the mean HS. Triangles: functional tissue radii; circles: geometric tissue radii.

The varying oxygen extraction rates from capillaries can be also be analyzed using the functional tissue radius of the supplied tissue territory around each vessel. The distribution of functional tissue radii (20.3 ± 6.3 μm and 19.9 ± 7.4 μm with the constant and random inflow value, respectively) has a larger spread than that of the geometric tissue radii (18.8 ± 4.5 μm) (Figures 5A–D). The difference between the standard deviations of the topological and geometric radii is significant (*p* < 10^−3^, *F*-test). Interestingly, the functional tissue radii only correlate weakly with the geometric tissue radii in the investigated CNs (*r* = 0.20, *p* = 0.040, *t*-test, Figure 5E). Albeit slightly smaller, the mean geometrical radii agree well with previous experimental data from the mouse cortex (Tsai et al., 2009; Sakadžić et al., 2014). The functional tissue radii were found to strongly correlate with the average HS in each vessel (*r* = 0.68, *p* = 1.8 × 10^−9^ in CN 1, *r* = 0.49; *p* = 5.0 × 10^−4^ in CN 2 with constant inflow HS, Figure 5F). The correlation is higher in CN 1 due to its larger size which causes a wider HS distribution and the presence of vessels with saturation <0.4. Similar figures were found with random inflow HS. The functional tissue radii strongly correlate also with RBC flow (*r* = 0.60) and significantly with RBC velocity (*r* = 0.47) and hematocrit (*r* = 0.45) in the simulations with constant inflow HS (Figures S4A–C). Finally, the functional tissue radii decrease with transit time and, to a lesser degree, with capillary path length (Figures S4D,E). These results directly support our modeling approach for capillary diffusive interaction where the supplied tissue radii are directly determined from the HS in the capillaries.

**Figure 6:**
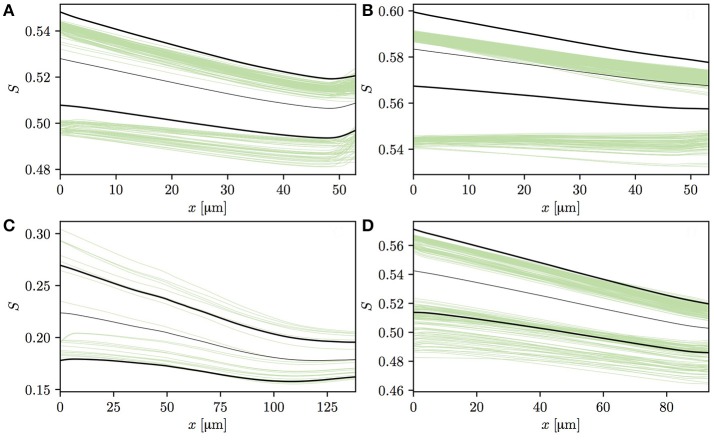
Profiles of HS in four selected vessels from CN 1 downstream of a converging bifurcation. Thin green lines: individual RBCs; thin black lines: mean HS $\bar{S}$; thick black lines: $\bar{S}\pm \sigma_{S}$. In panel **(A)**, the HS profiles slightly increase at the end of the capillary since it flows into another capillary with higher HS. In panel **(C)**, HS values are significantly lower since RBC flow in the parent vessels of the capillary is very low, hence the large upstream oxygen discharge.

Having analyzed the effects of capillary diffusive interaction, the evolution of HSH in individual vessels is now analyzed. The vessels in both CNs can be split into two categories based on the HS values at the vessel entrance. The first one consists of vessels that are downstream of a converging capillary bifurcation and may have a comparatively high HSH at their inlet. The second category is composed of vessels with low initial HSH, such as occur at the inflow boundary or in the absence of upstream converging bifurcations in the employed CNs. Figures 6A–D shows HS profiles in four selected capillaries in CN 1 that follow a converging bifurcation. In these vessels, the HSH σ_*S*_ significantly drops, similarly to the idealized scenario presented in Lücker et al. (2018). In two of these four capillaries (Figures 6A,B), many RBCs with lower initial HS almost do not release any oxygen, or even undergo an increase in *S*. In Figure 6A, the increase in *S* at the distal end of the capillary is caused by a converging bifurcation with a capillary with more highly saturated RBCs. The fact that RBCs from different vessels may overlap near bifurcations is likely contributing to this effect. However, these selected vessels display an evolution of HS which is very similar to that previously observed with alternating or uniform random values at the inflow (Lücker et al., 2018).

Microvessels with very low initial HSH display a different behavior. As shown in Figure S5, the standard deviation σ_*S*_ increases in these vessels. This phenomenon which was not observed in the idealized setups presented in Lücker et al. (2018) originates from rapid fluctuations of hematocrit that occur both in the employed discrete RBC simulations and in experiments (Chaigneau et al., 2003). For each RBC, the drop in HS along a capillary segment was correlated with the time difference between this RBC and the previous RBC. In three selected vessels, the standard deviation of *S* was increasing along the vessel, albeit at different rates, and a significant positive correlation was found (Figure S5). The observed correlation can be explained by transient oscillations in tissue Po_2_ caused by linear density fluctuations. For high spacings between RBCs, tissue Po_2_ undergoes a slight transient drop, which causes more oxygen to be extracted from the next RBC. Conversely, when two RBCs are close to each other, the second RBC is surrounded by tissue with elevated Po_2_ and releases less oxygen due to the lower Po_2_ gradient. Unlike our previous findings, these results suggest a mechanism that causes the HSH within single vessels to slightly increase.

**Figure 7:**
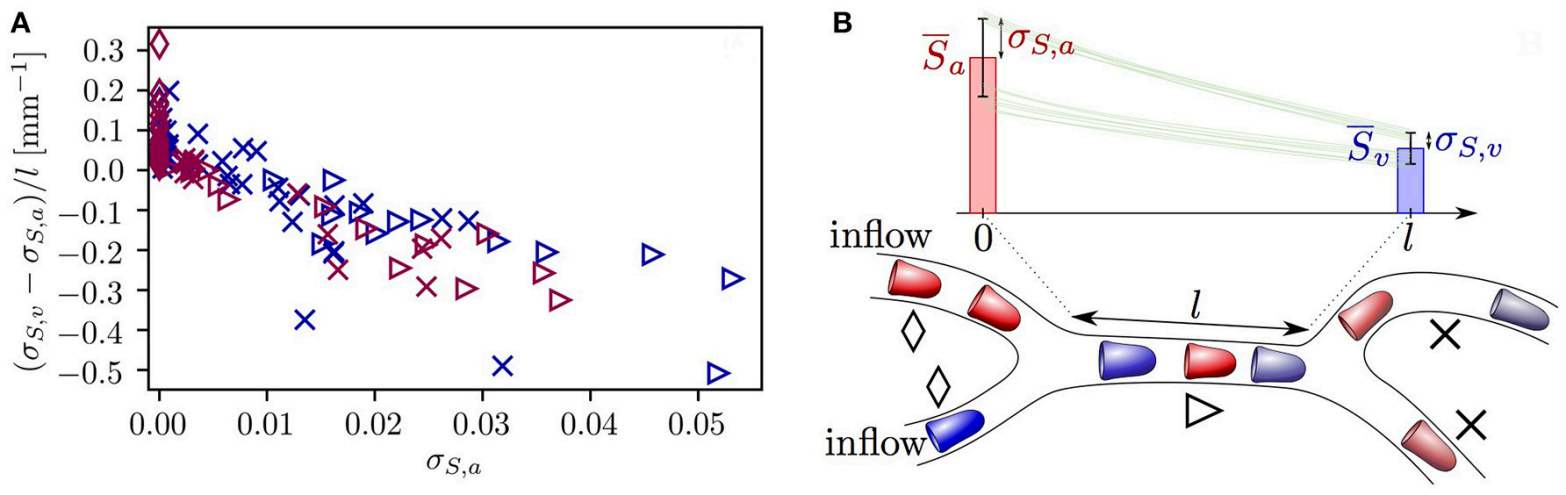
Evolution of the standard deviation of HS σ_*S*_ in each capillary in both reconstructed CNs. **(A)** Rate of change of σ_*S*_ across each vessel as a function of the inlet value of σ_*S*_ and the vessel type. Dark blue symbols: CN 1; purple symbols: CN 2. Diamonds: inflow vessels; triangles: vessels after a converging bifurcation; crosses: remaining vessels. **(B)** Explanatory sketch for the symbols used in **(A)**. The symbols show the three different vessel types. The bars illustrate the typical evolution of HS along a vessel located downstream of a converging bifurcation (mean ± SD). Green lines: representative profiles of HS in individual RBCs.

The microvessels that were selected so far exhibited opposing evolution of HSH, namely a reduction due to RBC diffusive interaction and an increase caused by hematocrit fluctuations. This raises the question whether one of these effects dominates the other. Figure 7 provides an overview of the evolution of σ_*S*_ in all vessels in both CNs. Most vessels with σ_*S,a*_ ≤ 0.005 undergo a slight increase in σ_*S*_. However, the standard deviation of HS decreases in all vessels with σ_*S,a*_ ≥ 0.01, many of which directly follow a converging bifurcation. Additionally, the reduction rate of σ_*S*_ along microvessels increases with σ_*S,a*_. This relation is a direct reflection of Equation (7) which states that dσ_*S*_/d*x* is negative and proportional to σ_*S*_. Therefore, although rapid fluctuations in hematocrit cause a non-negligible increase in HSH in some microvessels, larger heterogeneity is damped at a faster rate due to RBC diffusive interaction. This shows that the model predictions for RBC interaction also hold in realistic CNs.

## Discussion

Oxygen transport simulations were performed in reconstructed CNs from the mouse cerebral cortex to evaluate COSH and its relation with capillary transit time heterogeneity. We quantified the effects of diffusive interaction and found that it leads to a considerable homogenization of HS, as predicted by the models developed in Lücker et al. (2018). Capillary diffusive interaction contributes to this reduction to a greater extent than RBC diffusive interaction. The coefficient of variation of the HS drop across the networks was found to be smaller than that of capillary transit times in the simulations with constant saturation levels entering capillaries. Our analysis shows that the radius of the tissue region closest to a capillary only correlates weakly with the radius of the tissue region actually supplied by the vessel. Instead, HS and RBC flow were observed to be the strongest predictors for the radius of the supplied tissue territories. Thus, diffusive interaction provides a degree of robustness in the microcirculatory system's ability to achieve tissue oxygenation, despite multiple sources of HS heterogeneity.

The effects of diffusive interaction were compared using the distribution of HS at the distal ends of capillary paths. While the computational model with moving RBCs fully takes into account diffusive interaction, the differential equation model for HS enables turning off both RBC and capillary diffusive interaction when using the geometric tissue radii in Equation (3). In the employed CNs, diffusive interaction was observed to decrease COSH by 41 to 62% with both constant and random inflow values for HS. This allows to conclude that diffusive interaction considerably mitigates the occurrence of localized regions of hypoxia. These are more likely to occur when sources of COSH, such as CTH and variability in hematocrit, are too strong to be balanced by diffusive interaction, even if the average blood flow and HS are sufficiently high. Tissue regions with low baseline Po_2_ are particularly vulnerable to hypoxia following increased metabolic consumption, pathological perturbations or vascular malfunction (Devor et al., 2011; Sakadžić et al., 2014). Brain imaging modalities with spatial resolution ≥ 1 mm such as functional magnetic resonance imaging may fail to detect these regions due to their small size. However, coarse-scale measurements of cerebral blood flow, HS and CTH (Mouridsen et al., 2014) could be combined to quantify how likely fine-scale hypoxic tissue pockets are. This study demonstrates that any such modeling attempt needs to include diffusive interaction.

Capillary and RBC diffusive interaction could be isolated using the differential equation model for HS with geometric and functional tissue radii. In the simulations with constant HS at the inflow vessels, approximately 75% of the COSH reduction was due to capillary diffusive interaction. When using random inflow values, the importance of RBC diffusive interaction increases to 34% in CN 1 and 59% in CN 2 since more converging bifurcations with high HSH occur. Due to the bigger size of CN 1, we hypothesize that capillary diffusive interaction dominates RBC diffusive interaction in entire capillary beds of the cortical microvasculature. According to our models, capillary diffusive interaction is proportional to the capillary Po_2_ difference between neighboring capillaries. In other words, the diffusive nature of oxygen transport from capillaries to tissue reduces oscillations of capillary Po_2_ with high spatial frequency. Therefore, the spatial organization of capillaries with low or high transit times influences the strength of diffusive interaction. Our findings support the hypothesis that a capillary bed topology where low- and high-branching-order capillary segments are close to each other is beneficial for the homogenization of tissue oxygenation, as suggested by Sakadžić et al. (2014).

We now turn our attention to RBC transit times and their relation with HSH. The differential equation model (Equation 4) shows that the RBC transit time is a good predictor for the drop in HS across the CNs, which is reflected by the high associated correlation coefficients (*r* = 0.88 in CN 1, *r* = 0.81 in CN 2 with the constant inflow value). This agrees with the finding by Ellsworth et al. (1988) that path length variability substantially contributes to HS heterogeneity. However, the coefficient of variation of the HS drop is 28% (resp. 37%) lower than that of the transit times in CN 1 (resp. CN 2) in the simulations with constant inflow HS. Therefore, when properly normalized, CTH overestimates COSH at the scale of the investigated CNs. Thus, measurements of CTH using bolus tracking (Gutiérrez-Jiménez et al., 2016) or dynamic susceptibility contrast magnetic resonance imaging (Mouridsen et al., 2014) need to be scaled down to accurately reflect the heterogeneity of the HS drop across capillaries.

The tissue territories around each capillary were investigated by modeling them as cylinders and comparing their respective radii. The radius distribution of the tissue territories supplied by each vessel (functional radii) is significantly wider than that of the tissue regions defined using the nearest vessel (geometric radii) (Figure 5). Moreover, the standard deviation of the functional radii increases when the HSH is higher, as in the simulations with random saturation at the inflow. This shows that the functional radii adapt to reduce HSH by means of capillary diffusive interaction. Additionally, the correlation between functional and geometric radii is weak (*r* = 0.20, *p* = 0.040) and the functional radii correlate most with HS (*r* = 0.68 in CN 1) and RBC flow (*r* = 0.69 in CN 1). They were also found to decrease significantly with transit time and path length. These results are consistent with the decrease of supplied tissue radii along capillary paths obtained by Sakadžić et al. (2014) based on measured HS. The lower maximal functional tissue radii in our study (40 vs. 60 μm Sakadžić et al., 2014) can be explained by the limited size of the employed CNs. In the simulations with random inflow saturation, three capillaries were taking up oxygen from their surrounding tissue, which is consistent with the theoretical argument based on the Krogh cylinder proposed by Sakadžić et al. (2014).

The oxygen transport simulations in reconstructed CNs showed that rapid hematocrit fluctuations can cause the standard deviation of HS to slightly increase along vessels with very low initial σ_*S*_ (Figure S5). Our simulation results lead to the hypothesis that most capillaries display a small degree of heterogeneity in HS. Thus, setting the standard deviation σ_*S,a*_ to zero in inflow capillaries could be considered as a model artifact that artificially increases the number of vessels with low σ_*S,a*_ (Figure 7). However, despite these fluctuations, the heterogeneity of HS in individual capillaries was found to decrease in all vessels with a medium-to-large initial value of σ_*S*_. This leads to the conclusion that the reduction of HSH caused by diffusive interaction is stronger than its increase due to rapid hematocrit fluctuations.

The main limitation of this study is the size of the CNs employed in the oxygen transport simulations. Since our models are currently limited to capillaries, larger networks could not be simulated since they would contain arterioles and venules (pairs of penetrating arterioles are separated by a distance of 130 ± 60 μm, Nishimura et al., 2006). This prevents full capillary paths from being simulated and restricts both the development and the reduction of HSH. The mean transit path lengths in our simulations (0.17 mm in CN 1 and 0.12 mm in CN 2) are approximately half as long as those measured by Sakadžić et al. (2014). Similarly, the mean simulated transit times (0.17 s and 0.11 s in CN 1 and CN 2) are well below capillary transit times measured using bolus tracking (0.66±0.20 s, Gutiérrez-Jiménez et al., 2016). We hypothesize that simulations in larger CNs would exhibit a more important reduction of COSH by diffusive interaction due to the exponential decrease in HSH predicted by our models (Equations 5, 7). The extension of our models to arterioles and venules would provide a better quantitative insight into COSH and its relation with CTH.

In the absence of Po_2_ measurements in the studied CNs, the HS values in RBCs entering the computational domain needed to be chosen. To assess the influence of this boundary condition, the inlet values associated to each inflow vessel were either set to a constant value across the network or generated using a uniform distribution. Thus, our main conclusions about the reduction of COSH by diffusive interaction are robust with respect to the choice of boundary condition. However, our approach could be extended by a more systematic uncertainty quantification. For instance, Monte-Carlo simulations could be run with inflow values for HS generated using measurement-based distributions. This would enable a more robust quantification of the respective influence of capillary and RBC diffusive interaction.

Similarly, the inlet tube hematocrit was set to a fixed value due to the absence of experimental data. Effects of varying inlet hematocrit can be estimated according to the following arguments. The distribution of RBCs at diverging capillary bifurcations in artificial networks is not substantially affected by variations of the inlet tube hematocrit between 0.1 and 0.5 (Schmid et al., 2015, Figure 4). Also, the diffusive interaction time scale is relatively insensitive to changes in hematocrit for a given flow rate (Lücker et al., 2018, Figures 4, 7). Therefore, the reduction in COSH due to diffusive interaction is approximately independent of inlet hematocrit, for fixed flow rates. Under physiological conditions, an increase in hematocrit would generally result in a decrease in flow rate due to increased flow resistance. The resulting increase in capillary transit time would then enhance the reduction in COSH due to diffusive interaction.

Our computational model has the advantage of resolving both capillary and RBC diffusive interaction through the presence of individual moving erythrocytes. However, this comes at an elevated computational cost (~ 40 h on 24 cores for 10 s of simulated time in CN 1). The computational model by Goldman and Popel (1999) or the Green's function method (Secomb et al., 2000) can predict HS in CNs at lower cost, albeit without RBC diffusive interaction. The latter effect could be added to these models based on the evolution equation for HSH within a single vessel (Equation 7) and a suitable parameterization of the model coefficient *K*_*RI*_ characterized in Lücker et al. (2018). A computationally even cheaper alternative would be to employ the differential equation for HS (Equation 1) exclusively and not to compute explicitly tissue Po_2_. This would require the modeling of the supplied tissue radii (Equation 3). Our results suggest that the functional tissue radius around each vessel can be determined iteratively based on the HS of the vessels in the neighborhood of the current vessel.

The physiological importance of COSH should also be further investigated. The influence of disturbed capillary flow patterns on COSH should be studied to identify which kind of flow disturbances increases the risk of local hypoxia due to elevated COSH. Second, the mean and the heterogeneity of capillary outflow HS need to be simultaneously quantified to assess the effectiveness of oxygen supply to tissue. To achieve this, the coefficient of variation of capillary outflow HS could be a useful metric. Additionally, focusing on local hypoxia will likely lead to a moderate overestimation of the diffusive interaction time scale due to Hill's equation inaccuracy at low HS, as discussed in Lücker et al. (2018). Accuracy similar to that of Adair's equation could be obtained at low computational cost by using the Po_2_-dependent Hill coefficient introduced by Dash et al. (2015). The suggested independence of our main findings from the inlet tube hematocrit should also be confirmed by additional simulations.

This study confirms that the theoretical predictions made in Lücker et al. (2018) about the reduction of HSH by diffusive interaction also hold in CNs. In both studied CNs, COSH in the presence of diffusive interaction is approximately 40 to 60% lower than when simulated without. In most simulated cases, capillary diffusive interaction contributes more to the reduction of HSH than RBC diffusive interaction. The coefficient of variation of the HS drop across the CNs was found to be approximately one third lower than that of transit times. The previous conclusions can be rephrased as follows: (1) diffusive interaction leads to a strong reduction of small-scale HSH caused by CTH, heterogeneous hematocrit distribution and capillary spacings; (2) HSH can arise in the absence of CTH, mainly due to heterogeneous hematocrit distribution and to a lesser degree because of fast temporal hematocrit fluctuations; (3) CTH is closely related to COSH in CNs, but does not determine it. Therefore, diffusive interaction contributes to the microcirculatory system's ability to ensure tissue oxygenation by partially counterbalancing the sources of COSH. However, this ability may not be sufficient to compensate for increases in heterogeneity as may occur in cerebral small vessel disease. The study of capillary dysfunction in the cerebral microvasculature and its consequences on brain metabolism could substantially benefit from these insights.

## Author contributions

AL conceived of the study, implemented the algorithms, ran the simulations, interpreted the data and drafted the manuscript. TS contributed the initial idea for the study and revised the manuscript critically for conceptual content. MB performed the animal experiments and processed the blood flow data. BW and PJ conceived of the study and participated in its design.

### Conflict of interest statement

The authors declare that the research was conducted in the absence of any commercial or financial relationships that could be construed as a potential conflict of interest.

## Abbreviations

CN: capillary network
COSH: capillary outflow saturation heterogeneity
CTH: capillary transit time heterogeneity
HS: hemoglobin saturation
HSH: hemoglobin saturation heterogeneity
RBC: red blood cell.
