# Supplementary material for: The Relation Between Capillary Transit Times and Hemoglobin Saturation Heterogeneity. Part 2: Capillary Networks
Source: Front Physiol. 2018 Sep 21;9:1296. doi: 10.3389/fphys.2018.01296 (PMC6160581; doi:10.3389/fphys.2018.01296)
Supplement: Supplementary file 2 [file Data_Sheet_1.pdf]

Supplementary Material: The Relation between Capillary Transit Times and Hemoglobin Saturation Heterogeneity. Part 2: Capillary Networks

Adrien Lücker [1,*], Timothy W. Secomb [2], Matthew J. P. Barrett [3], Bruno Weber [3] and Patrick Jenny [1]

*Correspondence:
Adrien Lücker
luecker@ifd.mavt.ethz.ch

1 SUPPLEMENTARY VIDEO

Oxygen transport simulation in CN 1. HS is shown by the RBC color and P_{O_2} by the cutting plane color. The transparent tubes show the capillary lumen.

2 SUPPLEMENTARY METHODS

Supplementary Methods 1

Extension of the oxygen transport model to CNs. The oxygen transport model with moving RBCs (Lücker et al., 2014) was extended to capillary networks to enable the study of COSH in realistic geometries. The knowledge of the position and the velocity of individual RBCs is required by our algorithm. RBC trajectories through the capillary networks were obtained using the discrete RBC transport model by Schmid et al. (2017) that takes into account the Fåhraeus and Fåhraeus-Lindqvist effects, as well as phase separation at divergent bifurcations. The blood flow through the CNs was reconstructed based on sparse measurements of RBC velocities using the procedure described in the Supplementary Methods 2.

Since the time steps in the oxygen transport model are smaller than in the RBC transport model (Schmid et al., 2017), the computed RBC positions were linearly interpolated. RBCs were modeled as rigid cylinders with the axis tangent to the capillary centerline. The RBC meshes were obtained from a Cartesian mesh by removing all cells that were not contained in a cylinder with radius r_c and length L_{rbc}. Then, the mesh was scaled to have exactly the prescribed RBC volume V_{rbc}. The use of smooth cylindrical RBC meshes was found to yield the same results as with Cartesian-based meshes.

Since capillaries may have different cross sections, the RBC diameter was set to 80% of the lumen diameter. For capillary diameters between 4 and $8\,\mu\mathrm{m}$, this corresponds to an endothelial surface layer width between 0.4 and $0.8\,\mu\mathrm{m}$, which falls within the range of measured values (Pries et al., 1990). The discrete RBC transport model (Schmid et al., 2017) enforces that RBC centers are separated by a minimal distance $L_{\mathrm{rbc}} = V_{\mathrm{rbc}}/(\pi r_c^2)$, so the RBCs cannot overlap unless they are separated by a kink or a bifurcation.

This model solves the same governing equations for the oxygen partial pressure P and the HS S as in Lücker et al. (2014). The oxygen transport equation is given by

$$\frac{\partial \alpha P}{\partial t} + \boldsymbol{v} \cdot \nabla(\alpha P) = \nabla \cdot (D\alpha \nabla P) + C_0 f(P,S) - M(P), \tag{S1}$$

where $\boldsymbol{v}$ is the blood velocity and $C_0 = N_{\mathrm{Hb}} V_{\mathrm{mol,O_2}}$ is the product of the heme concentration and the molar volume of oxygen. In RBCs, the evolution of HS follows

$$\frac{\partial S}{\partial t} + \boldsymbol{v} \cdot \nabla S = \nabla \cdot (D_{\mathrm{Hb}} \nabla S) - f(P,S), \tag{S2}$$

where D_{Hb} is the diffusion coefficient of hemoglobin in RBCs.

Eq. (S1) was solved in a rectangular box that contains multiple capillaries and oxygen-consuming tissue. Eq. (S2) was solved in all moving RBCs that overlap with the box. The computational model (Lücker et al., 2014) allows for different diffusion and solubility coefficients in the RBC, plasma, endothelium and tissue. In capillary networks, this is done using an approach based on volume fractions, since the generation of a mesh that conforms with tortuous, branching capillaries would be very challenging. Instead, a Cartesian mesh was used to discretize the fixed domain. For each capillary, a tortuous cylinder following the centerline was constructed (Supplementary Figure S6). The cylinder was composed of an inner region for the lumen and an outer annulus for the endothelium. For each grid cell in the fixed mesh, the lumen and endothelium volume fractions γ_p and γ_w were obtained by computing its intersection volume with the reconstructed cylinder. At branchings between capillaries, the reconstructed cylinders may overlap. In this case, the contributions of each cylinder were added and the volume fractions were clamped so that their sum is at most one. The tissue volume fraction was then obtained by $\gamma_t = 1 - \gamma_p - \gamma_w$. At the beginning of each simulation, the diffusion and solubility coefficients for each grid cell without RBC influence were obtained as the weighted harmonic average of the respective coefficients in each region, where the weights are γ_p, γ_w and γ_t. Subsequently, at each time step, the obtained coefficient was corrected in grid cells that were overlapping with an RBC using the coefficient value in RBCs and the RBC volume fraction γ_c.

The velocity field in the blood vessels is required in Eq. (S1). To obtain it, for each vessel between nodes i and j, the RBC velocity $v_{\mathrm{rbc},ij}$ from the discrete RBC transport model was mapped to the matching capillary. This was done by tagging each grid cell with $\gamma_p > 0$ with its corresponding vessel index and assigning to it the velocity vector with magnitude $v_{\mathrm{rbc},ij}$ and direction tangent to the vessel centerline at the nearest point to the cell center. The resulting velocity field $\boldsymbol{u}_{\mathrm{mapped}}$ was generally not discretely divergence free because of the tortuosity of the vessels and the branchings, which would result in a violation of mass conservation. Therefore, the velocity field was corrected based on the Helmholtz-Hodge decomposition theorem which states that a smooth vector field $\boldsymbol{u}$ can be decomposed as the sum of a divergence-free velocity field $\boldsymbol{v}$ and an irrotational component as $\boldsymbol{u} = \boldsymbol{v} + \nabla\phi$. The velocity field $\boldsymbol{v}$ is obtained by applying the divergence operator, which results in the correction scheme

$$\nabla^2 \phi = \nabla \cdot \boldsymbol{u}, \tag{S3}$$

$$\boldsymbol{v} = \boldsymbol{u} - \nabla\phi. \tag{S4}$$

Eq. (S3) was solved on the submesh of the fixed mesh that is composed of all grid cells with $\gamma_p > 0$. Before solving for ϕ, the mapped velocity field $\boldsymbol{u}_{\mathrm{mapped}}$ was corrected at the boundary of the submesh so that the influx exactly matches the outflux on each connected component, which is required to fulfill

mass conservation. Finally, a homogeneous Neumann boundary condition was used for ϕ. Eq. (S3) was discretized using the central scheme and solved using the preconjugate gradient method.

Supplementary Methods 2

Flow reconstruction algorithm based on sparse measurements. Given a sparse set of RBC velocity measurements in a reconstructed CN, a matching blood flow field needs to be reconstructed for oxygen transport simulations to be run. In the networks used here, most boundary vessels are capillaries where the pressure is unknown. Therefore, the flow reconstruction method introduced by Gagnon et al. (2015) cannot be directly employed. Since RBC velocity measurements from longitudinal line scans are restricted to vessels in the microscope focal plane, the resulting flow reconstruction problem is generally underdetermined. To solve this issue, we introduce a cost function based on energy minimization and determine a pressure solution using optimization under constraints. The resulting pressure boundary conditions can subsequently be used in the discrete RBC transport model (Schmid et al., 2017) which provides RBC trajectories to our oxygen transport model with moving RBCs.

Consider a graph representation of a CN with a set of n_v vertices $\mathcal{V}$ and edges $(i, j) \in \mathcal{E}$ that represent blood vessels with associated lengths L_{ij} and diameters d_{ij}. The flow q_{ij} through a vessel (i, j) and the pressure difference between nodes i and j are related by the hydraulic resistance as

$$q_{ij} = \frac{p_i - p_j}{R^e_{ij}}. \tag{S5}$$

The effective resistance R^e_{ij} is related to the resistance obtained by Poiseuille's law by the in-vitro apparent viscosity $\mu_{\mathrm{vitro},ij}$ as

$$R^e_{ij} = \mu_{\mathrm{vitro},ij} \frac{128\mu_p}{\pi d^4_{ij}} L_{ij}, \tag{S6}$$

where μ_p is the plasma viscosity and was set to $1.2\,\mathrm{mPa\,s}$ (Késmárky et al., 2008). The expression for $\mu_{\mathrm{vitro},ij}$ is given in Pries and Secomb (2005) and depends on the vessel diameter and hematocrit. The determination of hematocrit is discussed below and its values can be assumed to be given for now.

The flow balance at each internal node j is enforced by

$$\sum_{i|(i,j)\in\mathcal{E}} \frac{p_i - p_j}{R^e_{ij}} = 0. \tag{S7}$$

Measurements of the RBC velocity $v^{\mathrm{meas}}_{\mathrm{rbc},ij}$ provide additional constraints. The corresponding flow is obtained by taking the Fåhraeus effect into account as follows:

$$q^{\mathrm{meas}}_{ij} = v^{\mathrm{meas}}_{\mathrm{rbc},ij} \frac{\pi d^2_{ij}}{4} \frac{H_{T,ij}}{H_{D,ij}}, \tag{S8}$$

where H_T and H_D are the tube and discharge hematocrit, respectively. The discharge hematocrit was computed from the tube hematocrit using empirical relations (Pries et al., 1990). Since some combinations of measurements can lead to a violation of flow balance, the vessels with associated measurements need to be split in two categories. For instance, if all edges that are adjacent to a certain vertex have prescribed RBC velocity values, the flows obtained with Eq. (S8) will not sum to zero in general. Denote by $\mathcal{E}_{\mathrm{comp}}$ the set of edges with compatible measurements that do not cause a violation of flow balance. A measurement

between nodes i and j yields the equation

$$\frac{p_i - p_j}{R^e_{ij}} = q^{\text{meas}}_{ij} \quad \text{for } (i,j) \in \mathcal{E}_{\text{comp}}. \tag{S9}$$

The treatment of incompatible measurements is discussed below.

The unknowns in the flow reconstruction problem are the pressure values at the n_v vertices in the network. Vertices can be split into $n_{v,\text{int}}$ internal nodes and $n_{v,\text{b}}$ boundary nodes. Flow conservation yields an equation for each internal node and each compatible measurement adds one more equation ($n_{\text{meas,comp}}$ in total). Therefore, if $n_{\text{meas,comp}} < n_v - n_{v,\text{int}} = n_{v,\text{b}}$, the problem is underdetermined, which occurred in both CNs employed here.

The following cost function is introduced to solve the underdetermination issue:

$$J(\boldsymbol{p}) = \omega_{\text{pow}} J_{\text{pow}}(\boldsymbol{p}) + \omega_{\text{v,avg}} J_{\text{v,avg}}(\boldsymbol{p}) + \omega_{\text{meas}} J_{\text{meas}}(\boldsymbol{p}), \tag{S10}$$

where $\boldsymbol{p}$ is the vector of pressure values at the nodes and the ω-coefficients have implied units. The first term represents the hydraulic power through the CN, namely

$$J_{\text{pow}}(\boldsymbol{p}) = \sum_{(i,j)\in\mathcal{E}} \frac{(p_i - p_j)^2}{R^e_{ij}}. \tag{S11}$$

This ensures that the pressure solution uses low hydraulic power, which expresses energy minimization. However, the use of this single term may cause vessels away from measurements to have zero or very low flow. To compensate for this effect, an additional term is introduced to guarantee an average RBC velocity in accordance with experimental data:

$$J_{\text{v,avg}}(\boldsymbol{p}) = (v_{\text{rbc,avg}}(\boldsymbol{p}) - v_{\text{rbc,ref}})^2, \tag{S12}$$

where $v_{\text{rbc,avg}}(\boldsymbol{p})$ is the length-weighted RBC velocity in the network for the pressure values $\boldsymbol{p}$ and $v_{\text{rbc,ref}}$ is a reference RBC velocity that can be chosen. The combination of the first two terms in Eq. (S10) with appropriate weights ensures that the obtained RBC velocities are neither too high nor too low (Figure 2).

The last term in Eq. (S10) addresses RBC velocity measurements that lead to a violation of flow balance. Let $\mathcal{E}_{\text{inc}}$ be the set of edges with measurements that are incompatible with flow balance. On these edges, the squared deviation between measurements and reconstructed flow is minimized using

$$J_{\text{meas}}(\boldsymbol{p}) = \sum_{(i,j)\in\mathcal{E}_{\text{inc}}} \left(q^{\text{meas}}_{ij} - \frac{p_i - p_j}{R^e_{ij}} \right)^2. \tag{S13}$$

The weights in Eq. (S10) were chosen to provide a satisfactory balance between the terms and set to $\omega_{\text{pow}} = 1$, $\omega_{\text{v,avg}} = 200$ and $\omega_{\text{v,avg}} = 50$ (there are only two actual degrees of freedom since the cost function can be arbitrarily rescaled by a multiplicative constant). The reference velocity $v_{\text{rbc,ref}}$ was set to $1.5\,\text{mm/s}$. Due to minimization of hydraulic power, the mean RBC velocities obtained with this method were close to $1.0\,\text{mm/s}$ (Table 1). This value can be adapted to obtain average RBC velocity values that match experimental observations. The minimization of $J(\boldsymbol{p})$ under the constraints given by Eq (S7) and

(S9) was performed using the implementation of sequential least squares programming in the scientific computing library SciPy (Jones et al., 2001–).

There remains to discuss the distribution of hematocrit in the CNs. An inflow tube hematocrit value $H_{T,\text{in}}$ of 0.25 was chosen as an initial condition. However, due to phase separation, the hematocrit distribution in CNs is not constant in general. When using the discrete RBC tracking model (Schmid et al., 2017) with the boundary pressure values $\boldsymbol{p}_b$ obtained from the minimization problem described above, the resulting steady-state hematocrit may differ from the initial hematocrit value. In turn, this affects the effective resistances R^e_{ij} (Eq. (S6)) and the agreement between the simulated flow and the measurements. Therefore, an iterative procedure is used where the steady-state hematocrit from the RBC transport model is fed back to the optimization procedure, which yields updated effective resistances. The flow reconstruction algorithm then provides a new pressure solution which is used as a boundary condition for the RBC transport model. The procedure is repeated until the resistances vary by less than 1%. This method is summarized in Algorithm 1 and was found to converge in both CNs. At each iteration, the RBC transport model was run for $1\,\text{s}$ of simulated time and the RBC positions from the previous iteration were used as an initial condition.

Algorithm 1: Flow reconstruction based on sparse measurements

compute $R^{e,0}_{ij}$ using $H_{T,\text{in}}$
$\epsilon_\text{rel} \leftarrow \infty$
$k \leftarrow 0$
while $\epsilon_\text{rel} < \texttt{rel\_tol}$ **do**
    $k \leftarrow k + 1$
    find $\boldsymbol{p}$ by minimizing $J(\boldsymbol{p})$ (S10) under the constraints (S7), (S9)
    run a discrete RBC transport simulation with $\boldsymbol{p}_b$ and $H_{T,\text{in}}$ at the boundary nodes over a given time interval
    compute $R^{e,k}_{ij}$ from the time-averaged tube hematocrit
    $\epsilon_\text{rel} \leftarrow \max_{(i,j)\in\mathcal{E}} \left| \frac{R^{e,k}_{ij}}{R^{e,k-1}_{ij}} - 1 \right|$
end while

REFERENCES

Gagnon, L., Sakadžić, S., Lesage, F., Mandeville, E. T., Fang, Q., Yaseen, M. A., et al. (2015). Multimodal reconstruction of microvascular-flow distributions using combined two-photon microscopy and Doppler optical coherence tomography. *Neurophotonics* 2, 015008. doi:10.1117/1.nph.2.1.015008

[Dataset] Jones, E., Oliphant, T., Peterson, P., et al. (2001–). SciPy: Open source scientific tools for Python. [Online; accessed 2017-11-24]

Késmárky, G., Kenyeres, P., Rábai, M., and Tóth, K. (2008). Plasma viscosity: a forgotten variable. *Clin Hemorheol Microcirc* 39, 243–246

Lücker, A., Weber, B., and Jenny, P. (2014). A dynamic model of oxygen transport from capillaries to tissue with moving red blood cells. *Am J Physiol (Heart Circ Physiol)* 308, H206–H216. doi:10.1152/ajpheart.00447.2014

Pries, A. R. and Secomb, T. W. (2005). Microvascular blood viscosity in vivo and the endothelial surface layer. *Am J Physiol (Heart Circ Physiol)* 289, H2657–H2664. doi:10.1152/ajpheart.00297.2005

Pries, A. R., Secomb, T. W., Gaehtgens, P., and Gross, J. F. (1990). Blood flow in microvascular networks. experiments and simulation. *Circ Res* 67, 826–834. doi:10.1161/01.res.67.4.826

Schmid, F., Kleinfeld, D., Blinder, P., Jenny, P., and Weber, B. (2017). Depth-dependent flow and pressure characteristics in cortical microvascular networks. *PLoS Comput Biol* 13, e1005392. doi:10.1371/journal.pcbi.1005392

SUPPLEMENTARY FIGURES

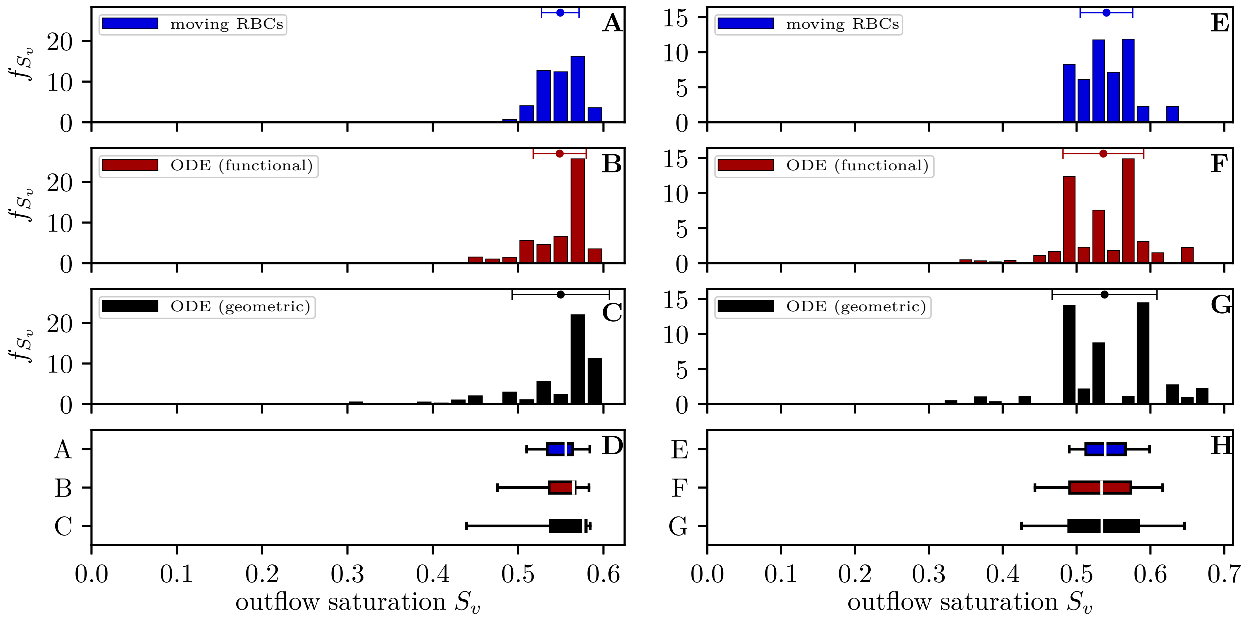

Figure S1. Distribution of the HS S_v at the distal end of capillary paths in CN 2. Left: constant inflow value; right: random inflow value. **(A, E)** computational model with moving RBCs; **(B, F)** differential equation model with functional tissue radii; **(C, G)** differential equation model with geometric tissue radii; **(D, H)** box plot with whiskers for the 5th and 95th percentile. The values from the differential equation model are weighted by the RBC flow in the distal capillaries. Error bars above the histograms: mean ± SD.

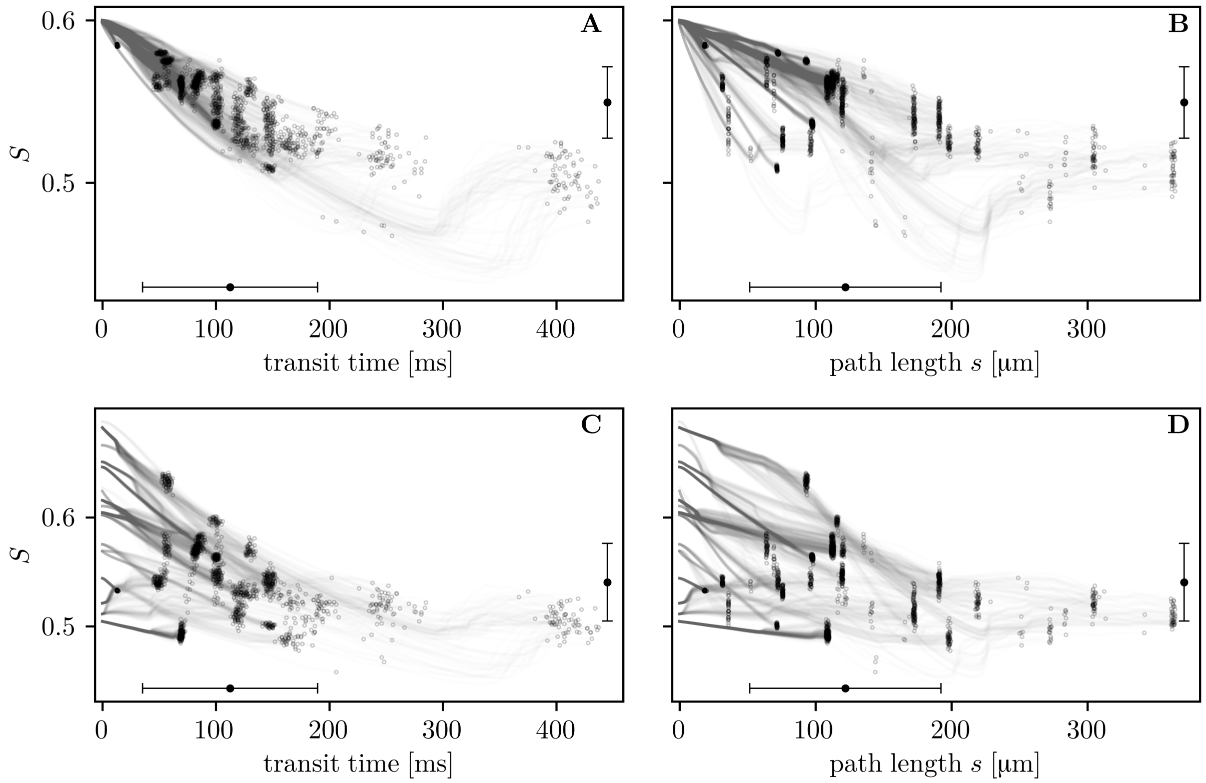

Figure S2. HS along individual RBC paths as a function of transit times **(A, C)** and transit path length **(B, D)**. The values are shown for simulations in CN 2 with constant **(A, B)** and random **(C, D)** inflow values. Circles: value in RBCs upon leaving the computational domain. Error bars: mean ± SD. The horizontal error bars pertain to entire RBC paths through the CN and the vertical error bars to outflow HS.

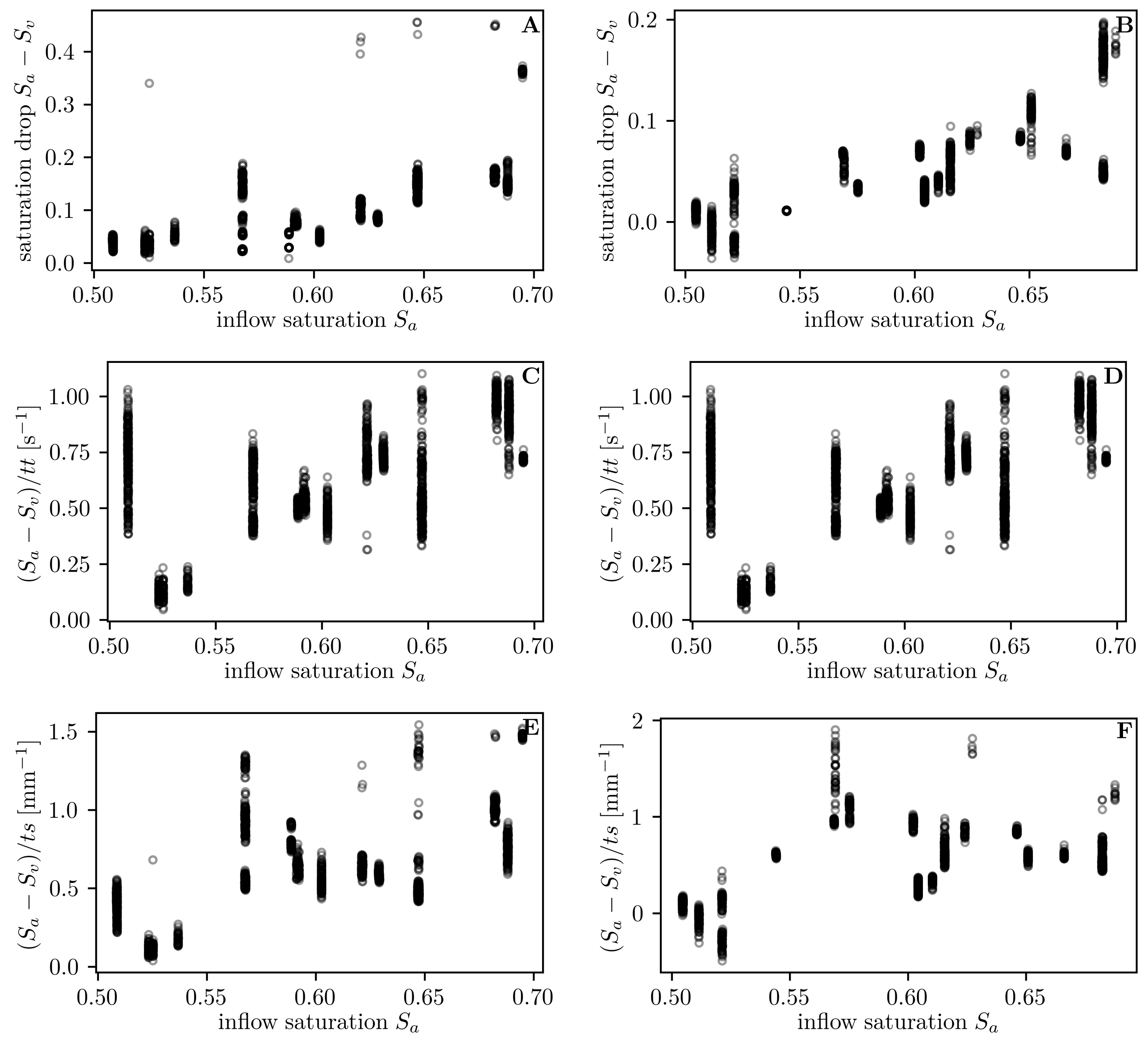

Figure S3. HS changes across CNs as a function of the random inflow saturation S_a. The respective Pearson's correlation coefficients r are given. **(A)** HS drop across CN 1 ($r = 0.70$); **(B)** HS drop across CN 2 ($r = 0.81$); **(C)** HS drop across CN 1 divided by the transit time ($r = 0.53$); **(D)** HS drop across CN 2 divided by the transit time ($r = 0.70$); **(E)** HS drop across CN 1 divided by the path length ($r = 0.53$); **(F)** HS drop across CN 2 divided by the path length ($r = 0.54$).

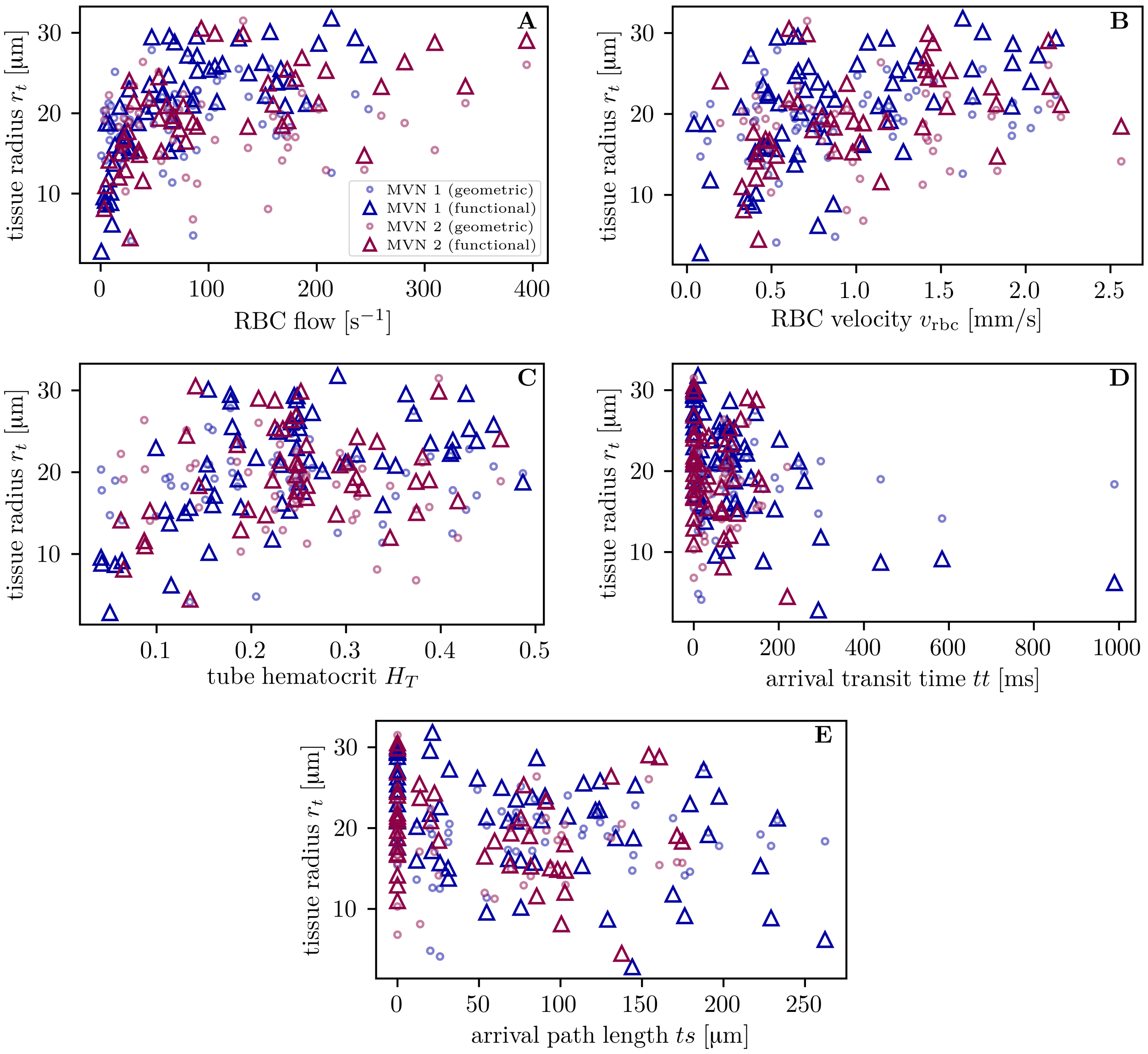

Figure S4. Tissue radii in the simulations with constant inflow HS. Triangles: functional tissue radii; circles: geometric tissue radii. Blue symbols: CN 1; purple symbols: CN 2. The respective Pearson's correlation coefficients are given. **(A)** RBC flow ($r = 0.60$); **(B)** RBC velocity ($r = 0.47$); **(C)** tube hematocrit ($r = 0.45$); **(D)** RBC transit time until entering the capillary ($r = -0.44$); **(E)** RBC path length until entering the capillary ($r = -0.33$).

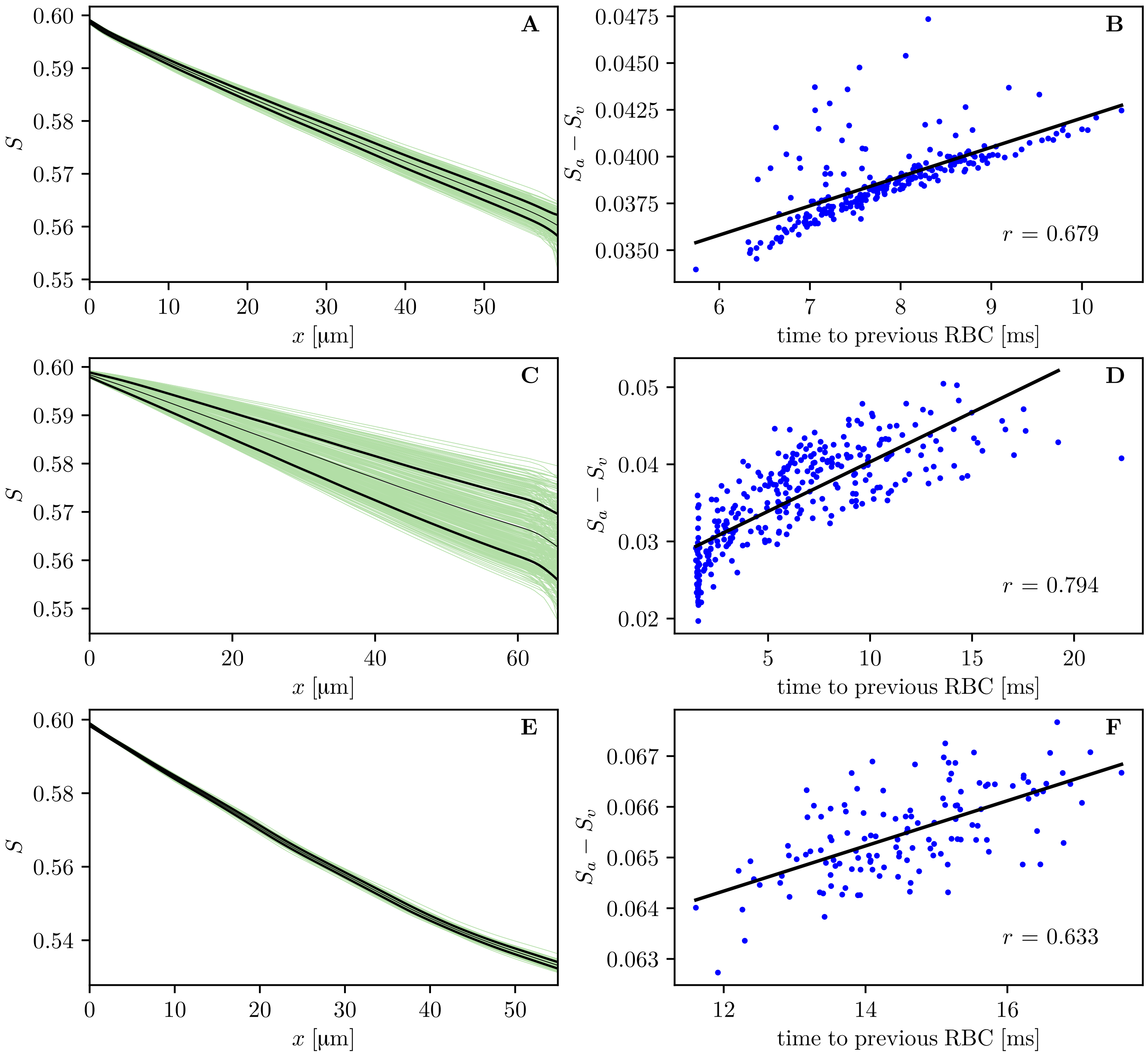

Figure S5. Profiles of HS in three selected inflow vessels in CN 1. **(A, C, E)** See legend of Figure 5. **(B, D, F)** Drop in HS for individual RBCs as a function of the time difference to the previous RBC that passed through the vessel; solid line: linear regression.

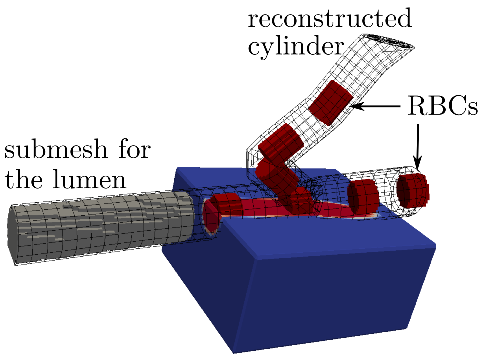

Figure S6. Meshes used in the model extension to capillary networks. The parallelepiped is a clipped part of the computational domain and the colors show values of the plasma volume fraction γ_p (red: 1; blue: 0). A fraction of the submesh for the lumen used by the velocity correction scheme is also shown.
